# Water Dipping of Auxin Coated Chrysanthemum Cuttings Confers Protection against Insect Herbivores

**DOI:** 10.3390/insects11110790

**Published:** 2020-11-12

**Authors:** Sanae Mouden, Kirsten A. Leiss, Henriette Uthe, Peter G.L. Klinkhamer

**Affiliations:** 1 Plant Sciences and Natural Products, Institute of Biology, Leiden University, 2300 RA Leiden, The Netherlands; p.g.l.klinkhamer@biology.leidenuniv.nl; 2 Business Unit Greenhouse Horticulture, Wageningen University & Research, Violierenweg 1, 2665 MV Bleiswijk, The Netherlands; kirsten.leiss@wur.nl; 3 Molecular Interaction Ecology, German Center for Integrative Biodiversity Research (iDiv), Halle-Gena-Leipzig, Deutscher Platz 5e, 04103 Leipzig, Germany; henriette.uthe@idiv.de; 4 Institute of Biodiversity, Friedrich Schiller University Jena, Dornburger-Str. 159, 07743 Jena, Germany

**Keywords:** indole-3-butyric acid, chrysanthemum cuttings, western flower thrips, leaf miner, *Frankliniella occidentalis*, *Liriomyza trifolii*, plant hormones, resistance, polyphenol oxidase

## Abstract

**Simple Summary:**

Cultivated chrysanthemums are one of the most economically important ornamental greenhouse crops worldwide. Classical breeding programs have mainly focused on improving aesthetic characteristics to meet the continuous increasing customer demands for new flower varieties. Consequently, commercial cultivars often lack insect resistance traits. Among the most important production constraints are biotic foes, in particular thrips and leaf miner infestations form a prominent hazard during its vegetative state. To maintain the desired aesthetic characteristics, clonal commercial propagation is aided by the use of auxin hormones for root promotion. This study aims to evaluate the potential of root promoting auxins in antiherbivore defenses. We demonstrate that water dipping of unrooted basal cut ends, coated with the commercial rooting hormone indole-3-butyric acid (IBA), conferred protection in chrysanthemum against thrips and leaf miner. Our findings add an interesting twist to the traditional role of auxins. We advocate a new twist of auxins beyond its traditional role in rooting in order to maximize plant yield by reducing herbivory through feasible, cost-effective water dipping treatments.

**Abstract:**

Auxins are commonly used for commercial propagation of chrysanthemums by stem cuttings. Recent studies imply that these root-promoting hormones also affect plant defense responses. The underlying motive of this study stems from the serendipitous observation that water dipping of auxin-coated cuttings beneficially affected thrips herbivory. Therefore, the primary objective of this investigation was to explore the role of indole-3-butyric acid (IBA) in relation to herbivore susceptibility in chrysanthemum. We observed contrasting findings concerning the physical presence of IBA and it’s role in promoting susceptibility of cuttings to thrips, which may in part be explained by the phenotypical variations of cuttings generated from mother plants. Nonetheless, we repeatedly demonstrated considerable protection, in some experiments up to 37%, against thrips and leaf miner upon water dipping of IBA-coated cuttings. Assessment of polyphenol oxidase activity (PPO), 14 days after dipping treatment, suggests that neither direct induction nor priming of plant defenses are involved. Future experiments aimed at understanding the early signaling events may help to explain the underlying mechanisms involved in conferring herbivore protection. We propose a dual role for auxins in early integrated pest management strategies to maximize plant development and minimize herbivory through feasible, cost-effective water dipping treatments.

## 1. Introduction

*Chrysanthemum morifolium* (Ramat) is a semi hardy herbaceous, perennial flowering plant and belongs to the family of Asteraceae (formerly known as Compositeae). Chrysanthemums are among the most important commercially grown greenhouse ornamentals worldwide and are extensively used as cut flowers and as pot plants [1,2,3]. Classical breeding programs have mainly focused on improving various characteristics to enhance ornamental values, including flower color, size and shape. Driven by such consumer needs, breeders are often under pressure to supply novel varieties within a restricted timeframe and with very specific choices, leaving few options for altering other agronomic traits. In addition to the limited gene pool, chrysanthemums are hexaploids and genetically highly heterozygous, thus complicating the development of resistant varieties and plant novelties at the same time [4]. Consequently, many commercial varieties often lack resistance traits to biotic and abiotic stresses.

Cultivars grown under greenhouse conditions are constantly challenged by a number of arthropod infestations among which susceptibility to the western flower thrips (WFT), *Frankliniella occidentalis* (Pergande), is one of the main constraints in the year-round production of quality chrysanthemums [5,6]. Thrips feed by piercing plant tissues with their needle-shaped mouthparts, causing two types of feeding damage [7]. Growth damage is caused by feeding on actively growing tissue whereas, feeding on older, expanded tissue causes cells to become filled with air. The latter, known as silver damage, significantly affects product appearance and hence reduces marketability. Insecticides have been a predominant management strategy, especially for high-value aesthetic ornamentals like chrysanthemum that require nearly zero level damage thresholds. However, once established in a greenhouse, control of thrips may be difficult to obtain due to their thigmotactic behavior and the limited number of active substances available [6]. Management of pests early in the production cycle is therefore, extremely important to prevent populations from building up to economically damaging levels. A second important pest species in ornamental greenhouses is *Liriomyza trifolii* (Burgess) [8]. Adult flies of the American serpentine leaf miner puncture leaves for feeding and oviposition. Feeding of larvae on the mesophyll leads to the formation of serpentine mines. Upon maturation, they exit the leaf to pupate in the soil [9]. Both larval and adult stages can cause a decrease in the photosynthetic area, facilitate entry of plant pathogens and adversely affect product appearance and yield.

Cultivars of commercially grown chrysanthemum are predominantly asexually propagated through vegetative terminal cuttings in order to generate genetically identical clones of the mother stock plants. Successful clonal propagation starts with adventitious root (AR) formation in stem cuttings and involves a large set of endogenous and exogenous factors [9,10]. Auxins, a class of plant growth regulating hormones, affect many crucial plant physiological processes and are key determinants of rooting propensity in cuttings [11,12,13]. Indole-3-buteryic acid (IBA) promotes rooting more efficiently, a feature partially due to its greater stability to light compared to auxin indole-3-acetic acid (IAA). Auxin formulations, applied to the basal cut end as dry-dip rooting hormone powder or as rooting solution (i.e., basal quick-dip), are commonly incorporated into propagation practices of commercial nurseries [14].

To mitigate pests early in the production cycle and prevent populations to build up to economically damaging levels, we aimed to evaluate the effect of various basal liquid dipping treatments to enhance herbivore resistance. We explored the use of plant derived compounds (i.e., secondary plant metabolites and plant hormones) as a prepropagation treatment to modulate or augment plant defenses. Surprisingly, we serendipitously observed dipping the basal cut ends of auxin-coated chrysanthemum cuttings in water caused a marked reduction in thrips-associated feeding damage. This observation led us to further explore the role of the growth hormone indole-3-buteryic acid (IBA) in relation to thrips defenses. In spite of the fact that the phytohormone auxin has classically been implicated in developmental processes, several studies demonstrate that auxin also affects a multitude of plant defense responses through complex interactions among multiple hormone pathways [15,16,17,18]. Extensive cross talk between auxin and jasmonic acid (JA) exists in both directions as these two hormones share many commonalities in their molecular mechanisms for hormone perception and signal transduction [19]. However, only a few reports are available regarding the exogenous effects of auxin on JA synthesis, which in addition, appear to be inconclusive. Auxin acts either synergistically or antagonistically with other hormones to trigger cascades of events leading to AR initiation and development [20]. Several lines of evidence indicate that auxins antagonize JA biosynthesis and signaling. External application of auxin suppressed herbivory-induced accumulation of jasmonates [21]. Likewise, a number of wound induced responses, including the expression of jasmonate-dependent proteinase inhibitor genes and vegetative storage proteins were negatively controlled by the level of auxin [22,23]. Down regulation of JA-responsive genes was also observed in auxin treated *Arabidopsis* plants [24,25]. Furthermore, auxin has been shown to induce expression of the JA signaling repressor JAZ1 [26]. Although all these experiments suggest an inhibitory effect of auxin on JA biosynthesis, contradictory results have been reported recently with auxins inducing the expression of JA biosynthetic genes [27,28]. Auxin also mediated increases in JA concentration whereas, mutants with no functional auxin receptors showed a reduction in JA amounts, suggesting a synergistic function of auxin and JA [29,30].

These results illustrate the complexity of auxin-induced changes and strongly imply their importance in plant stress responses, particularly toward biotic stresses. Numerous studies, mainly from the analysis of mutant phenotypes, have mostly demonstrated the involvement of auxins in disease development [15,30,31,32,33,34]. The changes incurred in plants as a result of these plant growth regulators may, therefore, likely affect plant–insect relationships. However, these effects have been poorly explored [16]. Given their importance in commercial plant propagation, the impact of auxin on insect herbivory deserves more attention if we are to understand their role as defense regulators.

In the present study, we aimed to investigate the effect of IBA-coating and water dipping in relation to insect resistance in commercial chrysanthemum cuttings. In this context, we hypothesized that chrysanthemum susceptibility to herbivory could be attributed to attenuation of induced defenses by exogenous auxins, possibly through its antagonistic effect on JA. Moreover, we explored plant defense responses triggered upon treatments by measuring the expression of the defense-related marker protein polyphenol-oxidase (PPO) and several defense- and growth-related phytohormones.

## 2. Materials and Methods

### 2.1. Plant Material and Growth Conditions

Chrysanthemum plants (*Chrysanthemum morifolium* Ramat) of the cultivar Baltica, which is susceptible to WFT, were used in all experiments and were kindly provided by Deliflor Chrysanten B.V. (Maasdijk, The Netherlands). Unrooted cold-stored chrysanthemum cuttings were imported from different production facilities located in Ethiopia (E) or Uganda (U). As a standard procedure for the production of commercial cuttings, the basal cut ends of freshly pinched cuttings were precoated with rooting powder by greenhouse workers, in order to stimulate root growth. Rooting hormone powder consisted of 0.4% indole-3 butyric acid in talc (Chryzoteck beige 0.4%) and was applied as a dry powder formulation. Unrooted chrysanthemum cuttings were approximately 5 cm long with three to four nodes. Commercial precoated cuttings were individually planted in plastic trays (4 cm × 4 cm × 6 cm) containing a 3:1 mixture of potting soil (Horticoop, Lentse Potgrond, The Netherlands) and vermiculite, premoistened to saturation using tap water. During the initiation phase of rooting, cuttings were covered with a transparent polyethylene plastic bag fitted over a plastic sliding tray (60 cm × 40 cm × 55 cm; Beekenkamp Verpakkingen B.V., Maasdijk, The Netherlands) to maintain humidity and prevent desiccation of cuttings. After a rooting period of 11 days, the cover was removed and cuttings were transplanted to plastic pots (Ø 11 cm; Pöppelmann, Germany) containing potting soil, 10 g/L vermiculite and 1.5 g/L osmocote slow release fertilizer (Scott, Scotts Miracle-Gro, Marysville, OH, USA, 15:9:11 NPK). Cuttings were grown in a climate room provided with 113.6 µmol photons m^–2^ s^−1^ of photosynthetically active radiation (PAR) and a light/dark cycle of 16/8 h at 20 °C and 70% relative humidity (RH) in a completely randomized design. Watering was applied every two days. At 14 days after planting, plants were randomly subjected to a non-choice whole plant thrips bioassay or sampled for chemical analyses as described below.

### 2.2. Non-Choice Whole Plant Thrips Bioassay

A non-choice whole plant bioassay was used to evaluate resistance against WFT [35]. Two-week old cuttings were individually placed in thrips-proof cages consisting of a Perspex cylinder (50 cm height, 20 cm diameter) closed at one end with a displaceable ring of nylon gauze of 120 µm mesh size. Plants were randomly placed in a climate-controlled growth chamber at a constant temperature of 25 °C, a photoperiod of 16L:8D (16 h Light:8 h Dark) and 70% RH. For infestation, adult thrips were collected in glass jars using a mouth-operated aspirator. Western flower thrips were obtained from a continuous mass-rearing on flowering plants of the susceptible chrysanthemum variety Euro Sunny and were maintained in a climate room at 25 °C and 70% RH. Twenty adult thrips, consisting of eighteen females and two males, were released inside the cage, simulating high-density infestations. Seven or 14 days after infestation, cuttings were visually inspected for thrips feeding damage, hereafter referred to as ‘silver damage’. Silver damage, expressed as damaged leaf area in mm^2^, was visually evaluated in all leaves. Whole plant cumulative silver damage of plants is presented in the graphs.

### 2.3. Non-Choice Whole Plant Leaf Miner Bioassay

Leaf miners, *Liriomyza trifolii* (Burgess), were obtained from a continuous mass rearing on a susceptible cultivar of chrysanthemum (ultra light) in a climate room provided with 16L:8D and 60% RH at 23 °C. In order to obtain pupae, infested plants containing third instar larvae were placed horizontally. One-day old flies were shortly anesthetized by CO_2_ and sorted by sex prior to release onto the plants. Two unmated males and females each were placed into a small cage with chrysanthemum cuttings. The adult leaf miners were allowed to deposit eggs on the plants for 24 h, after which they were removed from the cage. All plants, free of adult leaf miners, were then randomly moved to a clean climate room (16L:8D, 70% RH, at 25 °C). The total number of mines on the plants was counted 3 days after leaf miner release to prevent overlap in the formation of mines and, thus, allow for more accurate counts of individual mines. Plants were harvested by cutting them at the crown level and transferring them into individual Ziploc bags. Subsequently, these bags were placed in a climate chamber at 20 °C for pupae to develop. The number of pupae was scored one week after harvesting using a dissecting microscope (25×). Plants were carefully checked for remaining pupae in leaf tissue, which were indicated as non-emerged pupae.

### 2.4. Dipping Treatments

#### 2.4.1. Bioinsecticidal Dipping of Unrooted Cuttings

Bioinsecticidal cut end dips were evaluated as a pretreatment of unrooted chrysanthemum cuttings to enhance resistance against thrips. Treatments were based on the results of preliminary trials in the laboratory. The basal cut end (1 cm) of unrooted cuttings was individually dipped in an aqueous solution of beta-alanine at a final concentration of 100 mg/mL. Water dipping treatment was considered as the control whereas, a non-dipped group of unrooted cuttings was included as a negative control group. Furthermore, the effect of exogenously applied JA was included as a positive control. The JA stock solution (1.19 M) was prepared by dissolving 2.5 mg in 1 mL absolute ethanol, which, prior to use, was diluted to a final working concentration of 5 mM. Based on preliminary experimental results, a dipping time of 60 min was selected as an effective duration for JA treatment. Following the dipping treatment, cuttings were planted in a mixture of premoistened soil and vermiculite. Two weeks after rooting, chrysanthemum plants were subjected to a non-choice whole plant bioassay as previously described. Various dipping durations were evaluated (e.g., 30, 45 and 60 min) but pooled silver damage data were used for analysis.

#### 2.4.2. Water Dipping of IBA Precoated Cuttings in Relation to Insect Resistance

To investigate the role of auxins in chrysanthemum susceptibility and to explore the potential of water dipping treatments for resistance improvement, commercial cuttings precoated with IBA and non-coated control cuttings free of IBA were used. The basal end (1 cm) of the cuttings was individually dipped in water for 30, 45 or 60 min, whereas non-dipped cuttings served as a negative control (indicated as *t* = 0). Additionally, a positive control was included by dipping the basal cut ends for 60 min in a solution of 5 mM JA. The cuttings were immediately planted and randomly grown under conditions described above. Two weeks after rooting, chrysanthemum plants were subjected to a non-choice whole plant bioassay (thrips or leaf miner) or sampled for polyphenol oxidase measurements as described elsewhere. Plants were transferred to paper bags and dried in an oven at 50 °C for at least 3 days for measurements of plant dry mass.

#### 2.4.3. Standardization of IBA Applied Powder Formulation

The objective of the present experiment was to standardize hormone powder application in order to potentially reduce IBA-induced response variations among cuttings. Rooted Baltica cuttings, planted in small compost blocks of 64 cm^3^, were manually pinched to obtain stem cuttings of 5 cm in length. The full factorial experiment contained two factors, each with four levels. Main treatments were auxin application (no dip, 0.4 and 0.8% IBA and talc) and water dipping time (0, 30, 45 and 60 min). Indole-3-butyric acid was applied as a dustable talc-based powder formulation to basal cut ends. Talc powder, primarily composed of magnesium silicate (Mg_3_H_2_(SiO_3_)_4_; Sigma-Aldrich, St. Louis, MO, USA; particle diameter 1.9–2.3 μm), was used as an inert carrier for dry powder formulations and was homogeneously mixed with IBA at a final concentration of 0.4% and 0.8% (*w*/*w*). To satisfactorily adhere to the cut ends and to provide a medium of contact through which the hormone could enter plant tissues, the basal cut ends were prewetted with water. Subsequently, cut bases were dipped in 0.4% IBA, 0.8% IBA or talcum powder. To ensure precise application, the amount of powder applied to the cut base of the stem was standardized to approximately 150 mg. Excess powder was brushed off. Other sets of cuttings were untreated and served as a negative control. To mimic the proposed commercial application and to keep cuttings turgid, they were stored in sealed plastic bags under cold (4 °C) and dark conditions for a week prior to water dipping treatments. For water dipping treatments, unrooted cuttings were vertically placed in glass vials filled with a layer of water at room temperature, covering 1 cm of their basal cut end. Cuttings were individually dipped in water for 30, 45 or 60 min, whereas control cuttings were not dipped (*t* = 0 min). All cuttings were planted on the same day, after their respective basal water dip treatments, in a 3:1 potting mix consisting of potting soil with vermiculite. The diameter of the cutting insertion at the soil surface was standardized (5 mm) to minimize loss of IBA powder. Fifteen cuttings were treated as a sampling group of which ten cuttings from each treatment group were subjected to a non-choice whole-plant thrips bioassay and five cuttings per treatment were used for plant hormone analysis as described below. For statistical analysis, data for silver damage, hormone concentrations and dry mass were pooled (30–45 and 60 min) for each coating treatment.

#### 2.4.4. Comparing Dry-Dip Rooting Powder with Water-Based Rooting Solution

The efficacy of two different application methods for IBA was investigated to avoid potential confounding effects exerted by talc-powder. The basal cut end of commercially provided unrooted Baltica cuttings, precoated with IBA, was dipped in water for 30 min. The commercial rooting powder Chryzotek beige 0.4% contains 4000 parts of indole-butyric acid to a million parts of talc and thus, basal liquid dips were performed using concentrations equimolar to powder applied IBA. To this end, eight water soluble IBA tablets of 50 mg each (Rhizopon AA, Hazerswoude-Rijndijk, The Netherlands) were dissolved in 100 mL MilliQ water at RT to obtain a final concentration of 4000 ppm (Rhizopon). Cuttings, free of hormone powder were inserted in a floating mat of which approximate 1 cm of the basal cut end was dipped in the IBA suspension for 30 min. Water soluble IBA solution was constantly stirred to avoid precipitation. After dipping, cuttings were grown as previously described. Each treatment consisted of 15 replicates. Two weeks after rooting cuttings were subjected to a non-choice whole-plant thrips bioassay.

### 2.5. Effect of Dipping on Different Forms of Induced Resistance in Chrysanthemum

To evaluate whether reductions in thrips associated feeding damage operate in a JA-dependent manner, polyphenol oxidase activities were measured. Forty IBA-coated cuttings were dipped in water for 30 min. Control cuttings, precoated with IBA, were directly inserted in a premixture of soil and vermiculite (*n* = 40). Two weeks post treatment cuttings were randomly divided in two groups of 20 each and were either infested with thrips or sampled for PPO measurements (i.e., direct induction). To demonstrate whether the underlying mechanisms govern direct induction of defenses or prime for a potentiated response, cuttings were also sampled for PPO assessment after thrips infestation.

In parallel to this experiment, an additional 280 cuttings were subjected to the same treatments as above to determine whether defense responses were time-dependent. For this time course experiment, chrysanthemum cuttings precoated with IBA were subjected to control (no dipping) or water dipping (30 min) treatment. At 14 days after the start of the dipping treatments, all leaves were sprayed with 2 mL of 7.5 mM MeJA (Sigma-Aldrich) or treated with the corresponding mock solution consisting of 0.8% aqueous ethanol. JA-associated defenses were artificially induced using methyl jasmonate (MeJA) because inductive effects on PPO bioactivities were observed to be more consistent than herbivore induction. Ten cuttings from a given treatment were periodically sampled for PPO measurement at 0 (i.e., before hormone treatment), 12, 24, 36, 48, 72 and 168 h (7 days) after hormone application.

### 2.6. Polyphenol Oxidase (PPO) Activity

Polyphenol oxidase (PPO) activity was measured following the method of Stout et al. [36] with slight modifications. Two weeks after treatment (i.e., before infestation) or one-week post infestation, the third leaf from the bottom was sampled. Fresh leaf material was ground using a tissue lyser (Qiagen, Hilden, Germany) and stored at –80 °C until analysis for PPO activity. One hundred and fifty milligrams of fine powder was extracted with 1.25 mL ice-cold potassium phosphate buffer (0.1 M, pH 6.8) containing 7% (*w*:*v*) polyvinyl polypyrolidine. To this homogenate, 0.4 mL of a 10% solution of Triton X-100 was added. Plant extracts were vortexed for 2 min and centrifuged at 11,000× *g* for 10 min at 4 °C. The resulting supernatant was used directly as an enzyme source using chlorogenic acid as a substrate. The reaction mixture consisted of 5 µL enzyme extract and 1 mL of 2.92 mM chlorogenic acid dissolved in 0.1 M potassium phosphate buffer at pH 8.0. The rate in change of absorbance of this mixture was spectrophotometrically measured at 470 nm for one minute (UV-1800 UV–VIS spectrophotometer, Shimadzu Europe GmbH, Duisburg, Germany). PPO activities were calculated from the linear slope and were reported as changes in absorbance values per min per gram of fresh weight.

### 2.7. Hormone Analysis

To investigate the signaling pathways involved in auxin-mediated induction of defenses against WFT, we determined how coating and water dipping treatments influenced plant defense- and growth-related hormone levels. Two weeks after the initial dipping treatment, prior to thrips infestation, the basal third leaf was sampled for hormone analysis (*n* = 5). Analysis of jasmonic acid (JA), its biosynthetic precursor 12-oxo-phytodienoic acid (OPDA), jasmonic acid-isoleucine (JA-Ile), salicylic acid (SA), abscisic acid (ABA) and auxin (indole-3-acetic acid, IAA) were performed following previous procedures with some modifications [37,38]. Leaves were flash frozen in liquid nitrogen and stored at –80 °C until freeze-drying. Approximately 100 mg of frozen and homogenized leaf material was aliquoted in 2 mL Eppendorf tubes and extracted with 1 mL of methanol containing 40 ng of the phytohormone standards D_6_-ABA (Olchemin), D_6_-JA (HPC), D_6_-JA-Ile (HPC), D_6_-SA (Olchemin) and D_5_-IAA (Olchemin). Samples were vortexed for 10 min and centrifuged at 14,000 rpm for 10 min at 4 °C. Subsequently, the supernatants were transferred to new Eppendorf tubes and evaporated to dryness in a vacuum-concentrator at room temperature. The residue was dissolved in 20 µL 70% aqueous methanol (*v*/*v*) for 5 min using an ultrasonic bath, and centrifuged 5 min at 14,000 rpm. The supernatants were transferred to glass vials and then analyzed by LC–MS/MS. One microliter of each sample was injected onto a C_18_ Zorbax-Eclipse column (50 mm × 4.6 mm, 1.8 µm, Thermo fisher, Waltham, MA, USA). The mobile phase was comprised of LCMS-grade water (solvent A) and acetonitrile (solvent B), both containing 0.05% (*v*/*v*) formic acid. The program had a constant flow rate of 400 µL min^–1^ and consisted of 0–0.5 min 95% solvent A; 0.5–2.5 min 50% solvent A and 50% solvent B; 2.5–3.5 100% solvent B and 3.5–4.5 min 95% solvent A. The column temperature was set at 42 °C. The Triple Quad mass spectrometer (EVOQ Elite^TM^, Bruker Daltonics, MA, USA) equipped with a heated electrospray ionization probe was operating in the negative ionization mode. The ion spray voltage was set at −4500 eV, the cone temperature at 350 °C, the cone gas flow to 35 and the heated probe temperature to 300 °C. Nebulizing gas and probe gas flow were set at 60 psi, respectively. IAA was analyzed in the positive ionization mode (ion spray voltage 4500 eV). Phytohormones were measured by monitoring the transition *m*/*z* described in Supplementary Table S1 and were quantified using the signal of their corresponding internal standard, and expressed on the basis of fresh leaf weight. Data acquisition and processing was performed using Bruker Software MS Workstation (8.0).

### 2.8. Statistical Analyses

Normal distributions were confirmed by Shapiro–Wilk tests and homogeneity of variances were determined by Levene′s tests. Means were compared, where appropriate, using an unpaired Student’s *t*-test or one-way ANOVA followed by a Fisher’s least significance difference (LSD) post hoc test. When assumptions were violated, data transformations were performed to significantly reduce heteroscedasticity and normalize residuals. Alternatively, differences in means were analyzed using nonparametric alternatives. When the homogeneity of variance assumption was not met, differences in means were analyzed by a Welch’s *t*-test or a Welch’s ANOVA followed by Games–Howell’s post hoc. Upon violation of normality means were analyzed using non-parametric Mann–Whitney tests or by Kruskal–Wallis followed by Dunn’s test with Bonferroni correction for multiple comparisons.

Moreover, generalized linear models (GLMs) using linear distribution and identity link functions were used to analyze the effect of the dipping treatment, coating treatment and their interaction on silver damage symptoms, PPO activity, hormone concentrations and dry weight. Differences among groups were tested by Fisher’s LSD post-hoc test. Differences in all analyses were considered significant at *p* < 0.05. As an indication of which plant hormone variables would best predict variation in silver damage among treatments, multiple backward linear regression analysis was performed according to the Akaike information criterion (AIC). Variables were removed from the full model when the variance explained did not significantly improve the model (α = 0.05). Among the five generated models, the most significant model, retaining a set of two strong predictors of silver damage, were further analyzed using GLM. All statistical analyses were conducted with SPSS v. 24 software (IBM; SPSS Inc., Chicago, IL, USA).

## 3. Results

### 3.1. Effect of Bioinsecticidal Pretreatments on Chrysanthemum Susceptibility

**Figure 1:**
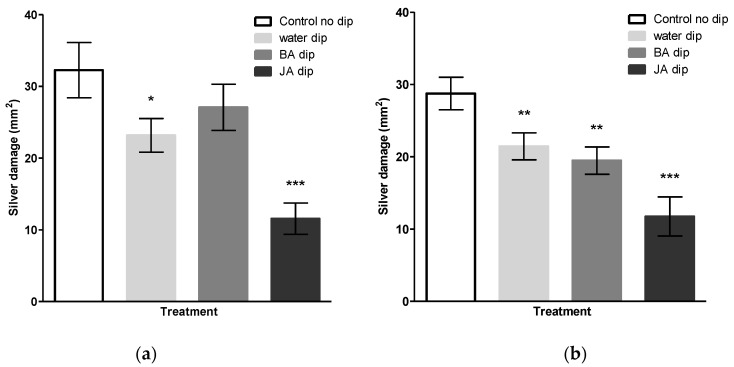
Effect of basal liquid dips on chrysanthemum resistance against western flower thrips (WFT). The basal portion of stem cuttings was dipped in 100 mg/mL of β-alanine (BA), water or 5 mM jasmonic acid (JA). Untreated, non-dipped, cuttings served as a control. All commercially provided cuttings were precoated with indole-3-butyric acid IBA (Chryzotek beige 0.4%). Two weeks post treatment, cuttings were infested with 20 adult thrips for a period of (**a**) 7 days or (**b**) 14 days. Silver damage symptoms were visually scored and expressed as damaged leaf area in mm^2^. Means for water dipping and β-alanine represent pooled cumulative silver damage of three dipping timepoints (30, 45 and 60 min). Data are presented as mean ± SEM. Asterisks indicate significant differences in comparison to non-dipped control as determined by an unpaired Student’s *t*-test. * *p* < 0.05, ** *p* < 0.01, *** *p* < 0.001.

The use of natural compounds with low risk profiles offer a simple and a cost-effective opportunity for sustainable pest management. Among them, β-alanine has reported confer resistance against WFT [39]. In preliminary experiments we observed that when the basal portion of chrysanthemum cuttings were dipped in a solution with β-alanine were more resistant to thrips, i.e., displayed less silver damage, than untreated cuttings of cuttings (Figure S1; *t*(7) = 2.149, *p* = 0.069). Therefore, we further investigated the potential of basal liquid dipping treatments, using aqueous solutions of β-alanine, as a possible strategy to enhance thrips resistance of chrysanthemum cuttings during their early vegetative stage. In comparison to non-dipped control cuttings, water dipping, but not β-alanine significantly reduced silver damage when cuttings were infested with thrips for one week (Figure 1a; *t*(23) = 2.137, *p* = 0.043). Furthermore, upon dipping in JA, thrips-associated feeding damage was markedly reduced by 65% relative to the control (*t*(18) = 4.680, *p* < 0.001). Following an infestation period of two weeks, the effect of β-alanine dipping treatment was highly significant (Figure 1b; *t*(46) = 3.623, *p* = 0.001) however, it did not differ significantly from water dipping with respect to a reduction in silver damage (Figure 1b). JA dip reduced silver damage by two-fold as compared to the control (*t*(28) = 5.439, *p* < 0.001). Taken together, these observations indicate that water dipping of IBA-coated cuttings was involved in enhancing resistance against thrips. Moreover, dipping treatments had no negative effect on the total dry mass of cuttings (*F*(7, 79) = 1.31, *p* = 0.257).

### 3.2. Water Dipping of IBA-Coated Cuttings Confers Protection against Thrips

**Figure 2:**
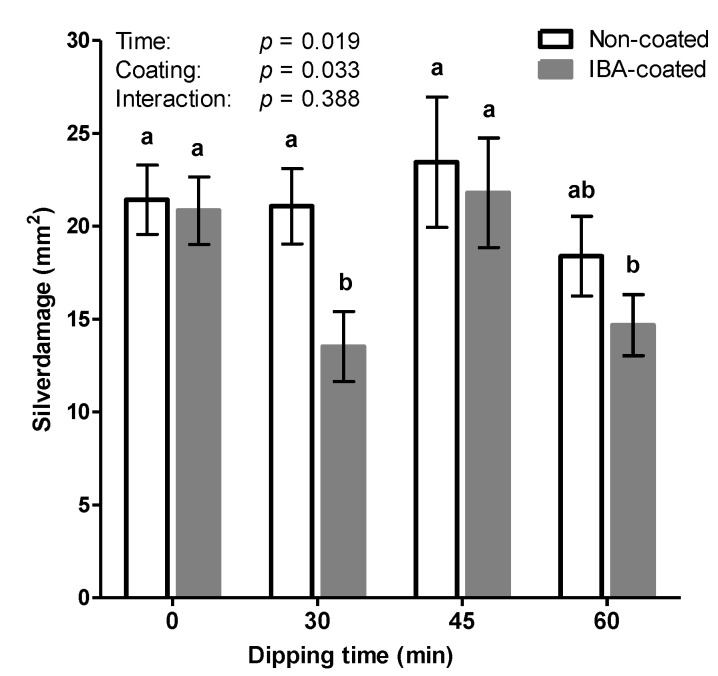
Effect of indole-3-butyric acid (IBA) coating and water dipping on chrysanthemum susceptibility against thrips. Basal cut ends (cv. Baltica), in the presence or absence of the powder formulated rooting hormone IBA, were dipped in water at various durations. Two weeks post treatment, cuttings were infested with 20 adult thrips for a period of one week. Cumulative silver damage data are presented as mean ± SEM (*n* = 10). Different letters denote significant differences among groups as determined by generalized linear model (GLM) followed by Fisher’s least significance difference (LSD) test (*p* < 0.05). The overall effects of dipping time, auxin coating and their interaction are indicated in the graph.

The observations of reduced silver damage upon water dipping gave rise to the hypothesis that auxins may exert an antagonistic interaction on the JA signaling pathway, and consequently, increase WFT susceptibility of cuttings commercially precoated with auxins. In order to evaluate the influence of auxin on thrips susceptibility, unrooted cuttings with and without IBA were infested with adult thrips after a rooting period of two weeks. Furthermore, we evaluated the effect of water dipping at three different time points namely; 0–30–45 and 60 min (Figure 2). Significant differences among treatments were observed, both for dipping time and auxin coating (GLM: Wald χ^2^ = 9.92, *p* = 0.019 and Wald χ^2^ = 4.55, *p* = 0.033, respectively). The time by coating interaction was not significant (GLM: Wald χ^2^ = 3.02, *p* = 0.388). Auxin coating of cuttings significantly affected silver damage symptoms however, mean comparisons within dipping time revealed there were no significant differences between IBA-coated and non-coated cuttings under control conditions (i.e., non-dipped cuttings at *t* = 0). Additionally, at 45 and 60 min no significant differences were observed between cuttings pretreated with IBA and untreated cuttings. Interestingly, however, water dipping of IBA treated cuttings significantly increased thrips resistance. The response to water dipping was independent of the time. After 30 and 60 min plants displayed significantly less silver damage. Silver damage symptoms were reduced up to 35% compared to plants grown from non-dipped IBA-coated cuttings. In contrast, in the absence of exogenous auxin coating, cuttings displayed no significant differences following water dipping.

### 3.3. Water Dipping of IBA-Treated Cuttings Reduced Pupae Emergence

The unexpected benefit of water dipping triggered our curiosity to further evaluate whether such an approach could improve resistance against other herbivores. Therefore, we evaluated the effect of water dipping treatments on celery leaf miner resistance, a second important pest insect species of chrysanthemum. Two separate experiments were performed to explore the role of IBA in leaf miner resistance. Firstly, we investigated the physical effect of the rooting hormone IBA on leaf miner resistance by comparing IBA-treated and non-treated cuttings (Figure 2). We observed that the mere physical presence of IBA did not influence susceptibility of chrysanthemums to leaf miners. Total number of mines (*U* = 74.5, *p* = 0.114) and the number of pupae (*U* = 91, *p* = 0.372) were not affected by exogenous application of IBA to cut ends (Figure S2A,B, respectively). The growth of cuttings, on the contrary, was significantly influenced by IBA. After two weeks of rooting, the number of leaves in cuttings pretreated with the rooting hormone IBA (9.60 ± 0.25) was significantly higher than the untreated control group (8.72 ± 0.23) (*t*(28) = −2.536, *p* = 0.017).

**Figure 3:**
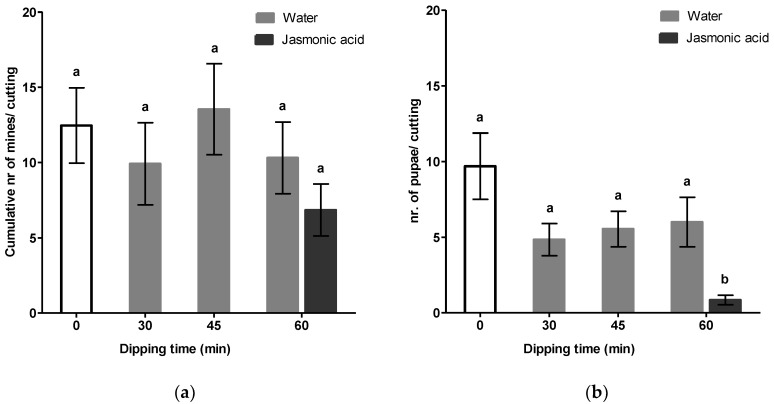
Effect of water dipping and jasmonic acid on leaf miner resistance (*Liriomyza trifolii*). Unrooted cuttings, precoated with indole-3-butyric acid (IBA), were dipped in water for various durations and in a solution of 5 mM jasmonic acid for 60 min. Two weeks post treatment, cuttings were individually caged and infested with four flies for 24 h. (**a**) Total number of mines were scored after 3 days, whereas the number of pupae were counted after one week (**b**). Data are represented as mean ± SEM, *n* = 13. Different letters indicate significant differences among treatments as determined by a (**a**) Fisher’s least significant difference (LSD) test or (**b**) Games–Howell at *p* < 0.05.

Additionally, in a second experiment we further assessed the effect of water dipping in the presence of the rooting hormone IBA on leaf miner (Figure 3). Group means of the total number of mines did not differ significantly (*F*(4, 59) = 1.08, *p* = 0.374). In contrast, the number of pupae was significantly affected by dipping (*F*(4, 28.52) = 10.59, *p* < 0.001). The amount of time cuttings were dipped in water had no effect on pupation. Post hoc comparisons revealed that non-dipped control cuttings and water dipped cuttings were similar to each other, but were significantly different from the JA treated group (Figure 3b). It is noteworthy, however, that the effect of water dipping becomes more evident when the ratio between emerged and non-emerged pupae was examined. Leaf miner larvae pupate in hibernacula within mines and emerged before or after leaf abscission.

**Figure 4:**
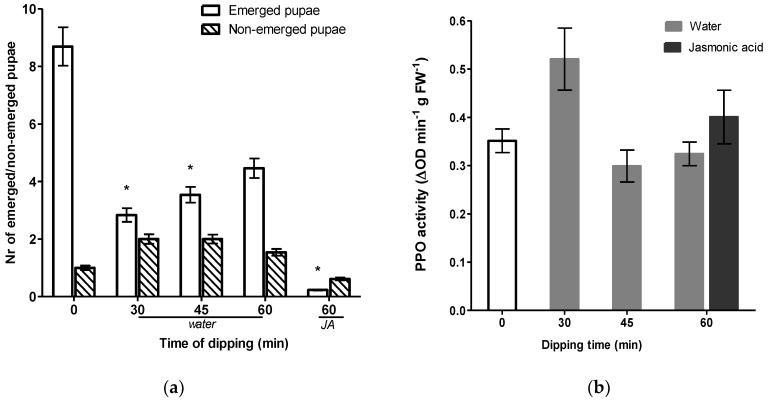
Effect of dipping on the leaf miner pupation and induction of polyphenol oxidase activity. Cut ends of chrysanthemum, pretreated with 0.4% IBA, were dipped in water for various durations and in a solution of 5 mM jasmonic acid for 60 min. Non-dipped cuttings, indicated at *t* = 0 in the graph, serve as a control. (**a**) Number of pupae emerging from chrysanthemum leaves and non-emerged pupae were scored after one week (*n* = 13). Asterisks indicate statistical significance in comparison to non-dipped control cuttings determined by Welch’s *t*-test (* *p* < 0.05). (**b**) Polyphenol oxidase activity (*n* = 8–10) was measured in the third leaf from the bottom prior to leaf miner infestation. Reciprocally transformed PPO data were analyzed by the Mann–Whitney test. Data shown are representative of means ± SEM.

Interestingly, upon further evaluation a significantly lower rate of emergence was observed for groups receiving a dipping treatment whereas, in comparison to the non-dipped control group, no differences for non-emerged pupae were observed (Figure 4a). The number of pupae successfully emerging from chrysanthemum leaves was significantly lower after 30 and 45 min of dipping *t*(15,27) = 2.55, *p* = 0.022 and (*t*(16,69) = 2.18, *p* = 0.044, respectively). For cuttings that were dipped in water for 60 min we also observed a reduction although not statistically significant (*t*(20,64) = 1.65, *p* = 0.115). Treatment of cut ends with JA significantly reduced emergence rate by 97% as compared to non-dipped control cuttings (*t*(12,08) = 3.93, *p* = 0.002). To investigate whether reduction in pupae emergence rates were related to induction of defenses, we assayed the activity of the defense-related protein polyphenol oxidase (PPO) by sampling leaf material prior to infestation (Figure 4b). An irregular pattern of PPO activity was observed in water and JA treatment groups. Activities of PPO were not significantly stimulated following dipping treatments suggesting that enhanced resistances were not explained by direct induction. Nonetheless, upon a dipping duration of 30 min in water, PPO levels were only marginally statistically enhanced in comparison to the non-dipped control (*U* = 19.0, *p* = 0.068).

### 3.4. Water Dipping Does Not Induce Nor Prime for Enhanced Defenses

**Figure 5:**
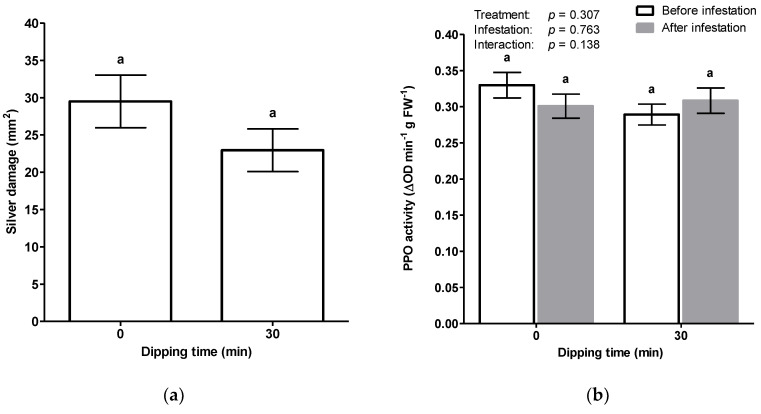
Effect of water dipping treatment on thrips-associated feeding damage (**a**) and polyphenol oxidase (PPO) activity (**b**). Cut ends of chrysanthemum cultivar Baltica, precoated with the powder formulated rooting hormone IBA (Chryzotek beige 0.4%), were dipped in water for 30 min. Two weeks post treatment, cuttings were infested with 20 adult thrips or sampled for PPO measurements before infestation. One week after thrips infestation, silver damage symptoms were visually scored and expressed as damaged leaf area in mm^2^ and subsequently sampled for PPO activity after infestation. Data in the graphs represent mean ± SEM of 20 individual cuttings. The main effects of dipping time, infestation and their interaction are indicated in the graph.

After evaluating the effect of auxin and dipping treatments on resistance to thrips and leaf miner, the most effective treatment was selected to further study the dynamics of induced resistance. We observed that efficacy of water dipping was most evident at 30 min. To demonstrate whether defense responses were induced directly or primed for potentiated expression upon thrips infestation, the activity of polyphenol oxidase was assayed by sampling leaf material before and after thrips infestation, respectively. Firstly, we investigated how water dipping of IBA-coated cuttings affected the induction of the JA-associated marker polyphenol oxidase and, concomitantly, the effect on resistance against WFT (Figure 5). In contrast to our previous observations, however, we found only a minor reduction in silver damage by water dipping and this was not significant (*t*(38) = 1.45, *p* = 0.156). The lack of enhanced resistance corresponds with the measured PPO levels. PPO activities were not stimulated upon water dipping treatment of cuttings (GLM, Wald χ^2^ = 1.05, *p* = 0.307). To further address whether water dipping primed plant defense, we assessed the induction of PPO activity under thrips infestation in control and water-dipped cuttings. Thrips infestation had no effect on the level of PPO (GLM, Wald χ^2^ = 0.09, *p* = 0.763).

**Figure 6:**
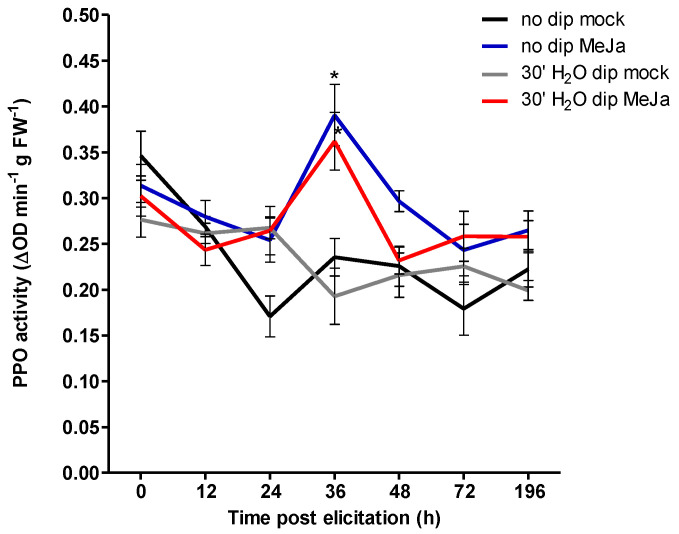
Effect of water dipping treatment on polyphenol oxidase activity. Comparative graph of PPO activity on a time course after MeJA elicitation in water dipped cuttings and non-dipped control cuttings. Basal ends of chrysanthemum cuttings (cv. Baltica), coated with the powder formulated rooting hormone IBA (Chryzotek beige 0.4%), were dipped in water at for 30 min or left untreated. Two weeks after rooting, cuttings were directly harvested for PPO analysis (*t* = 0 min) or sprayed with 7.5 mM MeJA to mimic thrips infestation. Mock treated plants served as control. PPO activity was measured in the third leaf from the bottom. Values represent the mean of 10 replicates with SEM. * Asterisks indicate significant difference at α = 0.05 as determined by an unpaired Student’s *t*-test.

In a further attempt to elucidate the role of auxin and water dipping treatments in establishing enhanced herbivore resistance, a time-course experiment was performed using MeJA for artificial induction of JA-associated defenses (Figure 6). In mock-treated plants PPO activities initially decreased and were then stabilized over time. PPO activities reached a peak level 36 h after methyl jasmonate (MeJA) application and then gradually declined at the end of the experiment. Exogenously applied MeJA significantly enhanced PPO activities by two-fold in water-dipped plants as compared to their corresponding mock-treated control (30 min H_2_O dip-mock; *t*(18) = 3.85, *p* = 0.001). Interestingly, the rate of PPO synthesis changed almost coordinately with non-water dipped plants and a similar trend was observed upon hormone treatment. PPO activity was directly responsive to exogenous MeJA treatment. Likewise, 36 h hours post elicitation, PPO levels were significantly amplified relative to the corresponding mock-control (*t*(18) = 3.95, *p* < 0.001).

### 3.5. Standardization of IBA Applied Powder

**Figure 7:**
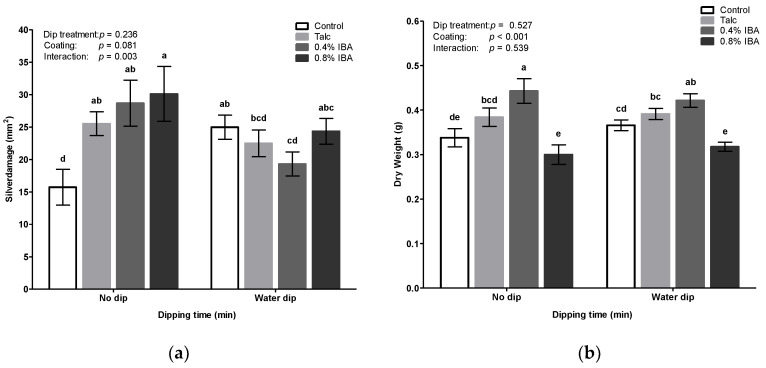
Effect of auxin formulation and duration of dipping on (**a**) thrips-associated feeding damage and (**b**) dry mass. Standardized powder treatments include prewetting of the cutting base in water prior to coating in talc or 0.4% and 0.8% indole-3 butyric acid (IBA). Chrysanthemum cuttings were individually placed in thrips proof cages and were exposed to adult thrips released at rate of 20 thrips per cutting. One week post infestation, silver damage symptoms were visually scored. Data are expressed as damaged leaf area in mm^2^ (means ± SEM). Means for water dipping represent pooled cumulative silver damages of three different dipping durations. Different letters denote significant differences among groups as determined by GLM followed by the Fisher’s LSD test (*p* < 0.05). The overall effects of dipping treatment, coating and their interaction are indicated in each graph.

Dipping treatments of chrysanthemum cuttings generally suffer from inconsistency. Many reasons can account for this situation among which a lack of standardized IBA formulation can be hypothesized. Indeed, within each batch of commercially provided unrooted cuttings, we observed considerable variation in the amount of IBA applied rooting powder at the basal cut ends (Figure S3). In view of this, the current experiment aimed to reduce response variations by regulating factors that may potentially influence absorption of IBA through the cut base. To this end, the loading amount of powder applied IBA at the cut base and duration of water dipping were systematically studied (Figure 7a). Silver damage did not significantly differ among various dipping treatment (GLM: Wald χ^2^ = 1.41, *p* = 0.236). Similarly, exogenous powder application neither affected silver damage symptoms (GLM: Wald χ^2^ = 6.73, *p* = 0.081). However, a significant interaction was observed between auxin treatment and dipping time (GLM: Wald χ^2^ = 13.91, *p* = 0.003). The least amount of silver damage was observed in non-coated cuttings under control conditions (i.e., control cuttings at *t* = 0 min). In the absence of a water dipping treatment, powder formulated hormonal application at the basal cut ends, irrespective of the type and concentration, significantly increased silver damage whereas, with increased duration of water dipping these negative effects were not observed.

These results contrast our previous observations, where no significant differences in silver damage were observed between IBA-coated and non-coated control cuttings (Figure 2). Upon water dipping, silver damage was significantly increased in control cuttings free of powder applied hormones while an opposite trend was observed for powder-coated cuttings. Notably, upon water dipping, IBA-coated cuttings, at a concentration of 0.4%, displayed considerably lower silver damage symptoms in comparison to non-dipped IBA cuttings. Silver damage symptoms were significantly reduced by approximately 33%. By contrast, the beneficial effect of water dipping was far less evident for cuttings treated with 0.8% IBA where water dipping only marginally reduced silver damage at 45 min. Furthermore, powder coating, but not water dipping, significantly influenced dry mass of chrysanthemum cuttings (GLM: Wald χ^2^ = 52.81, *p* < 0.001 for coating; Wald χ^2^ = 0.40, *p* = 0.527 for powder coating and Wald χ^2^ = 2.16, *p* = 0.539 for the interaction). Among hormone concentrations, application of 0.8% IBA to the cut base of the stem adversely affected dry mass (Figure 7b).

### 3.6. Hormonal Profiling

In an attempt to further elucidate factors underlying the phenomenon of enhanced resistance upon water dipping of IBA-coated cuttings, six major plant hormones were measured two weeks after dipping treatment (i.e., before thrips infestation). Multiple backward regression analysis was used to reveal the relative importance of plant hormones by eliminating variables with a low level of significance (Tables S2 and S3). The most significant model revealed that JA and its isoleucine conjugate, JA-ile, were significant predictors of silver damage among different treatments. These two variables predicted silver damage, F(2, 13) = 5.03, *p* = 0.024, *R*^2^ = 0.436. Subsequently, generalized linear models were applied to assess the main effects of cutting treatment and dipping time, and their pairwise interaction on JA and JA-ile (Table 1). The concentration of JA varied significantly among treatments (GLM: Wald χ^2^ = 6.75, *p* = 0.080 for dipping time; Wald χ^2^ = 39.50, *p* < 0.001 for cutting treatment and Wald χ^2^ = 29.90, *p* < 0.001 for the interaction). Jasmonic acid-isoleucine concentration, on the other hand, was only influenced by dipping time (GLM: Wald χ^2^ = 9.45, *p* = 0.024 for dipping time). Coating of cuttings with talc or IBA only marginally affected the concentration of JA-ile (Wald χ^2^ = 7.21, *p* = 0.065 for coating and Wald χ^2^ = 13.02, *p* = 0.162 for the interaction). However, reductions in silver damage were not accompanied by marked increases in JA nor by upregulation of its isoleucine conjugate and, thus, do not support a synergistic nor an antagonistic mode of action of IBA.

### 3.7. Comparing Dry-Dip Rooting Powder with Water-Based Rooting Solution

**Figure 8:**
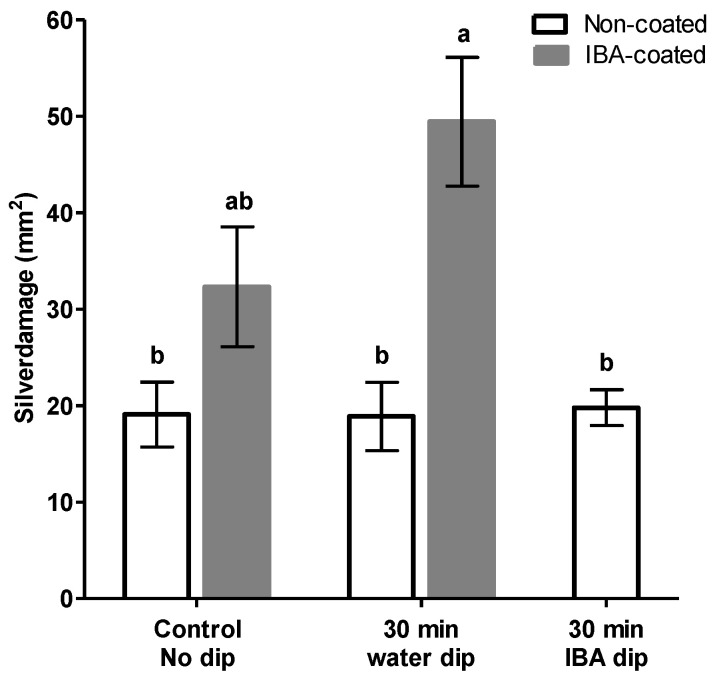
Effect of powder and liquid auxin formulations on silver damage. Unrooted IBA-coated (cv. Baltica) cuttings were dipped in water for 30 min. Non-coated cuttings were dipped in an equimolar concentration of 4000 ppm or water. Control cuttings did not receive a water dipping treatment. Bars (mean ± SEM, *n* = 13–15) represent the cumulative silver damage expressed as damaged leaf area in mm^2^. Different letters indicate significant differences among groups as determined by Kruskal–Wallis followed by Games–Howell multiple post hoc comparison (*p* < 0.05).

Due to possible confounding effects exerted by talc, we aimed to differentiate between the impact of IBA and that of the carrier chemical talc contributing to the absence of plant defense responses. In this context, basal liquid dips were performed using concentrations equimolar to powder applied IBA. Concomitantly, commercially provided cuttings precoated with 0.4% IBA were included in the experiment to compare the relative effectiveness of application and possibly the influence of IBA absorption through the cut base. Figure 8 shows that, in general, non-coated cuttings displayed the least amount of silver damage whereas, strikingly, water dipping of IBA-coated cuttings yielded the highest level of silver damage. The Kruskal–Wallis test showed that there was a statistically significant difference in silver damage symptoms between the different treatments groups, H(4) = 17.138, *p* = 0.002. However, we observed no evidence that auxin supplementation enhanced reduced feeding symptoms, with the exception of water dipped IBA-coated cuttings, were equal across all groups. Under control conditions, when cuttings did not receive a water dipping, an increase in silver damage was observed in IBA-coated cuttings (32.3 ± 6.2), although this effect was not significantly different from non-coated cuttings (19.1 ± 3.4).

## 4. Discussion

The European agriculture is at an important juncture and in a period of tremendous change, where the acronym IPM (integrated pest management) is endorsed as the future paradigm for crop protection [40]. With reduced availability of chemical interventions, established cropping systems are highly vulnerable to disruption [6]. Biopesticides, particularly plant-based formulations, appear at the horizon as an attractive strategy for sustainable control of pest insects [41,42]. Bioinsecticidal dips of cuttings, carried out as immersion treatments, primarily focus on postharvest disinfestation for trade and export purposes to manage insect populations prior to shipment. These interventions, i.e., quarantine, and strategies allow one to control pest populations prior to entering the production cycle [43,44] but do not provide any protection during the production. Therefore, we evaluated the effect of various basal liquid dipping treatments as a prepropagation treatment to enhance defenses against two economically important pest insects of *Chrysanthemum*, WFT and leaf miner, with the final aim to reduce reliance on chemical pesticides early in the production cycle.

Many plant secondary metabolites display interesting bioactivities, which either act directly as a result of their insecticidal properties or indirectly by mediating induced defense responses. Among the defensive metabolites, β-alanine has frequently been implicated in resistance against herbivores of numerous taxa, including western flower thrips [39]. In preliminary experiments we observed that when the basal portion of cut ends was dipped in an aqueous solution of β-alanine, silver damage symptoms were reduced by 36% as compared to non-dipped chrysanthemum cuttings.

Subsequently, in seeking to optimize the dipping treatments of chrysanthemum cuttings, we serendipitously observed that silver damage was remarkably lower in water dipped cuttings (Figure 1). Since the base of commercial chrysanthemum cuttings was covered with a rooting power containing auxin, this outcome led us to explore the mechanistic crosstalk of auxins in relation to plant–insect defenses. While jasmonates are generally recognized as the prominent hormone in plant defense against herbivores, recent studies reveal that auxins may also have a key role in modulating defense processes [45]. The assumption of a dual role of auxins is supported by several lines of evidence. A number of studies have demonstrated an inhibitory effect of exogenously applied auxins on JA-biosynthesis [21,22,23,24,25,26]. Exogenous application of the growth regulating auxin hormone IBA could have attenuated thrips resistance by exerting an antagonistic effect on the JA-signaling pathway. As such, a direct prediction of our first hypothesis is that the physical presence of IBA on stem cuttings promotes thrips susceptibility whereas, removal of externally applied hormone powder, by water dipping treatments, could mitigate the antagonistic effect of auxin, which supports our serendipitous observation. This hypothesis was tested by performing water dipping treatments in the presence and absence of the rooting hormone IBA. When cuttings received no water dipping treatment, we observed no significant differences in silver damage between IBA-coated and uncoated cuttings (Figure 2), suggesting that auxins do not play a direct role in promoting susceptibility of cuttings to thrips. Intriguingly, water dipping treatments in the presence of IBA-coating was shown to significantly reduce silver damage symptoms whereas, non-coated cuttings displayed no significant differences in thrips damage following water dipping.

The simplicity and beneficial effect of water dipping triggered our curiosity to further evaluate whether such an approach could improve resistance against multiple herbivores. Subsequently, we evaluated the effect of dipping treatments on celery leaf miner resistance, a second important pest insect species of *Chrysanthemum*. In agreement with our thrips data, it is clear that the mere physical presence of IBA did not explain susceptibility of *Chrysanthemum* cuttings to *Lyriomyza trifolii*. Furthermore, we observed that water dipping had no direct effect on the number of mines (Figure 3a). Although seemingly lower, no significant differences were detected in total pupation but the number of pupae successfully emerging from leaves was significantly lower after water dipping (Figure 4a). The most prominent effect was observed in JA treated cuttings. Cut ends dipped in JA significantly reduced the emergence rate of pupae by 97%. Induced resistance may result from a direct activation of defense mechanisms, including increased basal levels of defense-related enzymes such as polyphenol oxidase (PPO), which serve an antinutritive role by reducing the digestibility of the dietary protein [31,45,46,47]. In order to determine whether this enhancement was associated with JA-dependent defense traits, we measured the induction activities of PPO. Although significant reductions in the number of emerged pupae were observed, none of the dipping treatments stimulated PPO activity. Surprisingly, JA dipping did not induce PPO levels either.

Rather than direct induction of defenses, it is plausible that dipping treatments of auxin-coated cuttings primed for enhanced defense reactions. The priming defense processes remain dormant until herbivore infestation and reflects a cost-effective approach by which the plant can avoid expending energy under low pest pressure [47,48]. To establish whether enhanced resistance in *Chrysanthemum* was associated with priming of the plant defensive capacity, a comparative study was undertaken. Firstly, we compared thrips-mediated induced responses in IBA-coated *Chrysanthemum* cuttings following a 30 min water dipping treatment. Again, we found a reduction of silver damage after water dipping but, this time it was not statistically significant (Figure 5a). Subsequently, as a marker of plant defense responses, the activity of PPO was measured two weeks post treatment (i.e., before thrips infestation) and one week post thrips infestation (Figure 5b). We observed no induction of PPO activities upon 30 min of water dipping. Furthermore, no significant differences were observed between thrips-infested and non-infested *Chrysanthemum* cuttings.

In parallel, a separate time-course experiment was performed to assess water dipping effects on artificially induced plant defense responses triggered by exogenous application of methyl jamonate (MeJA). The time course of PPO levels in cuttings treated with MeJA peaked after 36 h for both the non-dipped and water dipped plants suggesting that water dip treatments in the presence of IBA did not directly induce nor prime JA related defenses or possibly operates independently from proteinaceous mediators such as PPO. However, the lack of effect could be explained by the time gap following dipping treatment and PPO measurements. While exogenous auxin treatment has been shown to increase PPO activity [49], peak values of PPO activity induced by exogenous auxin appear to occur at different time points, depending on the plant species and hormone treatment. For example, in 1-naphthylacetic acid treated cuttings of hybrid aspen, PPO levels significantly increased 6 days after treatment [50] whereas, Zhang and coworkers [49] reported a PPO peak value 45 days after planting IBA treated stem cuttings of *Malus hupehensis*. As such, the current experimental set-up does not allow us to draw a conclusive picture of underlying mechanisms and indicate the necessity of follow up studies to demonstrate whether induction or priming of plant defenses are involved.

Considering earlier conflicting results concerning defense responses of *Chrysanthemum* cuttings to water dipping, we emphasized the importance of standardizing hormone application to reduce batch variations in hormone application (Figure S3). In line with our previous results, we observed that water dipping significantly reduced silver damage in cuttings treated with 0.4% IBA as compared to its corresponding non-dipped control (Figure 7). Multiple backward linear regression analysis revealed that differences in silver damage among *Chrysanthemum* treatments could be explained in a model comprising JA and JA-isoleucine. However, it did not yield a consistent pattern explaining the relation of hormones associated with reductions in thrips-associated feeding damage. Importantly, these results should be interpreted in the context of spatial and temporal limitations. Hormonal concentrations were measured prior to thrips infestation and thus, two weeks post treatment, such measurements do not necessarily encompass hormonal changes induced by treatment of cuttings.

Despite the extensive standardization efforts, dipping and coating treatments continued to suffer from a high level of irreproducibility in thrips responses. Surprisingly, IBA-coating elevated the susceptibility of cuttings to thrips, as manifested by significant increases in silver damage at *t* = 0 min (Figure 7). Possibly, the talc powder used in commercial rooting hormones acted as a confounder. Talc, a clay mineral composed of hydrated magnesium silicate (Mg_3_H_2_(SiO_3_)_4_), is a functional carrier in many agricultural products and is extensively used as an inert chemical for active premixed ingredients. Talc is a strongly hydrophobic and hence, the naturally water-repellent talc particles can form a barrier when they envelop other particles. This could reduce the evaporation and uptake of water by preventing the formation of hydrate bridges, which is used to enable longer storage periods [51]. Hence, the level of water stress in cuttings, as a result of the talc-based coating, may have negative consequences for herbivores, especially for those that rely on high tissue water content and turgor (i.e., osmotic) pressure. Moreover, contrary to our earlier observations, water dipping of control cuttings free of powder applied hormones negatively affected silver damage. Symptoms were markedly increased by 37% relative to non-dipped cuttings (Figure 7), which, likewise, may be explained in relation to their water status. One of the main constraints in large-scale production of unrooted cuttings is the time delay between excision of cuttings from stock plants and insertion into a rooting environment. Once a stem is cut from its mother plant, its nutrient and water supply is lost. Traditionally, herbivorous insects are thought to exhibit enhanced performance on water-stressed host plants due to induced changes in plant physiology, largely through their effects on nitrogen availability [52]. However, adaptive plant responses to simultaneously occurring stresses (abiotic and biotic) are far more complex and compromise a fine-tuned network of hormonal signaling cascades mediated by abscisic acid [53,54], which require more in-depth early analysis. Alternatively, phenotypical differences in susceptibility may also result from the fact that auxins play a key role in regulating senescence [30,55] and can contribute to cross-regulatory mechanisms between leaf senescence and plant immunity [56]. We suggest that future studies focusing on early, hormonal signaling events, i.e., by performing time course experiments during rhizogenesis, could help to elucidate underlying molecular mechanisms in order to demonstrate the importance of auxins in antiherbivore defenses.

Finally, in an effort to identify and avoid possible confounding effects of talc-powder, basal liquid dips were performed using concentrations equimolar to powder applied IBA. We detected no evidence that IBA supplementation by liquid dips improved thrips resistance as silver damage. Strikingly, upon water dipping, IBA-coated cuttings displayed significantly more silver damage as compared to non-coated cuttings. The large variation within and between experiments and the contrasting results leads to our second remark and concerns the status of the mother stock, which, in addition to the water status of plant cuttings, may play an important role. An explanation of the apparent disparity of plant responses to thrips-associated feeding patterns may be the phenotypic heterogeneity of plant cuttings. We speculated that the physiological status of stock plants is key to explaining the discrepancy in the variable and inconsistent performances of thrips on generated cuttings. Maternal life history traits may affect the progeny, i.e., generated stem cuttings, and thus, despite being clonal in nature, cuttings can display different response patterns when facing the same infestation conditions. First and foremost, discrepancies in experimental results could be linked to the origin of cuttings and collection time of cuttings. Experiments conducted with cuttings from Ethiopia were harvested from mother plants in week 49 (December 2017) whereas, in the latter experiment for IBA standardization, pruned cuttings from mother plants grown in a greenhouse located in Uganda differed by one week in harvest date (week 27 and week 28; July 2018). In addition to regional or climatic influences, it is important to note that growth conditions among these commercial greenhouses are optimized for each production site and, as such, may for example affect the nutritional status of stock plants (S. Kos, pers. comm.). This lends additional support to the contention that phenotypic heterogeneity involves genotype by environment interactions. Other hypothetical possibilities that influence physiological responses include; but are not limited to, substrate, cutting position along the canopy structure of the stock plant, age and the health of mother plants.

## 5. Conclusions

### Auxins: A Dual Tool for Integrated Pest Management

In summary, we demonstrated that water dipping of IBA-coated cuttings repeatedly reduced herbivory, by thrips and by leaf miner although, results were highly variable among repeated experiments. This adds an interesting twist to auxins as a successful strategy to the horticultural toolbox of *Chrysanthemum* propagators. Separation of IBA and talc may imply a possible confounding effect of the carrier chemical. Future experiments aimed at understanding the early signaling events, including hormonal signaling networks, from a more holistic perspective may help to explain the underlying molecular mechanisms involved in conferring protection against herbivores. A possible explanation of the anomalies appeared to hinge on the large phenotypical variation in cuttings generated from stock plants and warrants the necessity to improve intra- and intervariability and reproducibility. Nonetheless, our results were of translational value and provide an interesting foundation for future applied studies to determine whether commercial auxin formulations provide protection over a broader range of herbivores and to evaluate its compatibility with other IPM tactics such as the use of natural enemies.

## Figures and Tables

**Table 1:** Hormonal profiling upon cutting treatments. Hormone concentrations are expressed as a mean in ng/mg freeze dried leaf material ± SEM (*n* = 5).

Cutting Treatment		Dipping Time	Hormone Concentrations			
			Ja-Ile		JA	
Treatments	Control	0 min	0.11 ± 0.05	bcde	58.91 ± 8.61	cdef
		30 min	0.05 ± 0.01	de	50.31 ± 7.07	fg
		45 min	0.04 ± 0.01	de	84.88 ± 8.30	ab
		60 min	0.09 ± 0.05	cde	77.28 ± 7.02	abd
	Talc	0 min	0.33 ± 0.12	ab	82.55 ± 7.00	ab
		30 min	0,04 ± 0.01	de	75.89 ± 5.26	abc
		45 min	0.41 ± 0.26	a	92.40 ± 10.12	a
		60 min	0.06 ± 0.04	cde	75.43 ± 10.15	abc
	IBA 0.4%	0 min	0.08 ± 0.03	cde	69.83 ± 4.80	bcd
		30 min	0.21 ± 0.16	abcde	68.74 ± 11.59	bcde
		45 min	0.20 ± 0.03	abcde	61.11 ± 6.46	cdef
		60 min	0.02 ± 0.01	e	40.33 ± 5.27	g
	IBA 0.8%	0 min	0.26 ± 0.09	abcd	53.70 ± 5.79	defg
		30 min	0.12 ± 0.06	bcde	48.14 ± 5.41	fg
		45 min	0.29 ± 0.11	abc	51.32 ± 3.40	efg
		60 min	0.14 ± 0.05	bcde	61.42 ± 7.34	cdef
GLM	Coating	Wald χ^2^	7.21 *p* = 0.065		39.50 *p* < 0.01	
	Dipping time	Wald χ^2^	9.48 *p* = 0.024		6.75 *p* = 0.08	
	Interaction	Wald χ^2^	13.02 *p* = 0.162		29.90 *p* < 0.01	

Abbreviations: indole-3-butyric acid (IBA); jasmonic acid (JA); JA-isoleucine (JA-Ile). Different letters denote significant differences among treatment groups as determined by generalized linear models (GLMs) followed by LSD at *p* < 0.05.

## Supplementary Materials

The following are available online at https://www.mdpi.com/2075-4450/11/11/790/s1. Figure S1: Efficacy of bioinsecticidal dips on chrysanthemum resistance against western flower thrips (WFT), Figure S2: Effect of exogenously applied indole-3-butyric acid (IBA) on celery leaf miner (*Liriomyza trifolii*) resistance, Figure S3: Substantial variation in the amount of hormone applied rooting powder within a batch of 50 commercially provided Baltica cuttings, Table S1: Transitions or specific pair of *m/z* values associated to the precursors and fragment ions of the analytes measured by LC/MS, Table S2: Backward multiple regression models of independent predictors of variation in silver damage, Table S3: Model summary of the backward elimination multiple regression analysis.

## References

[1] Fletcher J.T. (1992). Disease resistance in protected crops and mushrooms. Euphytica.

[2] Machin B. (1996). Cut flower chrysanthemum production. Grower Guide 4.

[3] Xia Y., Deng X., Zhou P., Shima K., Teixeira da Silva J.A., da Silva J.A.T. (2006). The world floriculture industry: Dynamics of production and markets. Floriculture, Ornamental and Plant Biotechnology: Advances and Topical Issues.

[4] da Silva J.A.T., Shinoyama H., Aida R., Matsushita Y., Raj S.K., Chen F. (2013). Chrysanthemum biotechnology: Quo vadis?. Crit. Rev. Plant Sci..

[5] Guldemond J.A., Tigges W.T., de Vrijer P.W.F. (1994). Host races of *Aphis gossypii* (Homoptera: Aphididae) on cucumber and chrysanthemum. Environ. Entomol..

[6] Mouden S., Sarmiento K.F., Klinkhamer P.G.L., Leiss K.A. (2017). Integrated pest management in western flower thrips: Past, present and future. Pest Manag. Sci..

[7] De Jager C.M., Butôt R.P.T., De Jong T.J., Klinkhamer P.G.L., Van Der Meijden E. (1993). Population growth and survival of western flower thrips *Frankliniella occidentalis* Pergande (Thysanoptera, Thripidae) on different chrysanthemum cultivars. J. Appl. Entomol..

[8] Van Dijk M.J., Hermans C., De Jong J., Van Der Meijden E. (1992). The impact of environmental conditions on survival of the leaf miner Liriomyza trifolii on Chrysanthemum cultivars. Proceedings of the 8th International Symposium on Insect-Plant Relationships.

[9] De Jong J., Van De Vrie M. (1987). Components of resistance to *Liriomyza trifolii* in *Chrysanthemum* morifolium and *Chrysanthemum pacificum*. Euphytica.

[10] Druege U., Franken P., Hajirezaei M.R. (2016). Plant hormone homeostasis, signaling, and function during adventitious root formation in cuttings. Front. Plant Sci..

[11] Druege U., Hilo A., Pérez-Pérez J.M., Klopotek Y., Acosta M., Shahinnia F., Zerche S., Franken P., Hajirezaei M.R. (2019). Molecular and physiological control of adventitious rooting in cuttings: Phytohormone action meets resource allocation. Ann. Bot..

[12] Bellini C., Pacurar D.I., Perrone I. (2014). Adventitious roots and lateral roots: Similarities and differences. Annu. Rev. Plant Biol..

[13] Pacurar D.I., Perrone I., Bellini C. (2014). Auxin is a central player in the hormone cross-talks that control adventitious rooting. Physiol. Plant..

[14] Blythe E.K., Sibley J.L., Tilt K.M., Ruter J.M. (2007). Methods of auxin application in cutting propagation: A review of 70 years of scientific discovery and commercial practice. J. Environ. Hortic..

[15] Kazan K., Manners J.M. (2009). Linking development to defense: Auxin in plant–pathogen interactions. Trends Plant Sci..

[16] Erb M., Meldau S., Howe G.A. (2012). Role of phytohormones in insect-specific plant reactions. Trends Plant Sci..

[17] da Costa C.T., de Almeida M.R., Ruedell C.M., Schwambach J., Maraschin F.D., Fett-Neto A.G. (2013). When stress and development go hand in hand: Main hormonal controls of adventitious rooting in cuttings. Front. Plant Sci..

[18] Brauer E.K., Rocheleau H., Balcerzak M., Pan Y., Fauteux F., Liu Z., Ouellet T. (2019). Transcriptional and hormonal profiling of *Fusarium graminearum*-infected wheat reveals an association between auxin and susceptibility. Physiol. Mol..

[19] Pérez A.C., Goossens A. (2013). Jasmonate signalling: A copycat of auxin signalling?. Plant Cell Environ..

[20] Lakehal A., Bellini C. (2019). Control of adventitious root formation: Insights into synergistic and antagonistic hormonal interactions. Physiol. Plant.

[21] Baldwin I.T., Zhang Z.P., Diab N., Ohnmeiss T.E., McCloud E.S., Lynds G.Y., Schmelz E.A. (1997). Quantification, correlations and manipulations of wound-induced changes in jasmonic acid and nicotine in *Nicotiana sylvestris*. Planta.

[22] Kernan A., Thornburg R.W. (1989). Auxin levels regulate the expression of a wound-inducible proteinase inhibitor II-chloramphenicol acetyl transferase gene fusion in vitro and in vivo. Plant Physiol..

[23] DeWald D.B., Sadka A., Mullet J.E. (1994). Sucrose modulation of soybean Vsp gene expression is inhibited by auxin. Plant Physiol..

[24] Rojo E., Titarenko E., Leon J., Berger S., Vancanneyt G., Sanchez-Serrano J.J. (1998). Reversible protein phosphorylation regulates jasmonic acid-dependent and –independent wound signal transduction pathways in *Arabidopsis thaliana*. Plant J..

[25] Liu J., Wang X.J. (2006). An integrative analysis of the effects of auxin on jasmonic acid biosynthesis in *Arabidopsis thaliana*. J. Integr. Plant Biol..

[26] Grunewald W., Vanholme B., Pauwels L., Plovie E., Inze D., Gheysen G., Goossens A. (2009). Expression of the Arabidopsis jasmonate signalling repressor JAZ1/TIFY10A is stimulated by auxin. EMBO Rep..

[27] Tiryaki I., Staswick P.E. (2002). An Arabidopsis mutant defective in jasmonate response is allelic to the auxin-signaling mutant axr1. Plant Physiol..

[28] Fattorini L., Veloccia A., Della Rovere F., D’Angeli S., Falasca G., Altamura M.M. (2017). Indole-3-butyric acid promotes adventitious rooting in *Arabidopsis thaliana* thin cell layers by conversion into indole-3-acetic acid and stimulation of anthranilate synthase activity. BMC Plant Biol..

[29] Grossmann K., Rosenthal C., Kwiatkowski J. (2004). Increases in jasmonic acid caused by indole-3-acetic acid and auxin herbicidesin cleavers (*Galium aparine*). J. Plant Physiol..

[30] Nagpal P., Ellis C.M., Weber H., Ploense S.E., Barkawi L.S., Guilfoyle T.J., Hagen G., Alonso J.M., Cohen D., Farmer E.E. (2005). Auxin response factors ARF6 and ARF8 promote jasmonic acid production and flower maturation. Development.

[31] Robert-Seilaniantz A., Grant M., Jones J.D. (2011). Hormone crosstalk in plant disease and defense: More than just jasmonate-salicylate antagonism. Annu. Rev. Phytopathol..

[32] Čarná M., Repka V., Skůpa P., Šturdík E. (2014). Auxins in defense strategies. Biologia.

[33] Zhang H., Tan X., Li L., He Y., Hong G., Li J., Sun Z. (2019). Suppression of auxin signalling promotes rice susceptibility to Rice black streaked dwarf virus infection. Mol. Plant Pathol..

[34] Djami-Tchatchou A.T., Harrison G.A., Harper C.P., Wang R., Prigge M., Estelle M., Kunkel B.N. (2020). Dual role of auxin in regulating plant defense and bacterial virulence gene expression during *Pseudomonas syringae* PtoDC3000 pathogenesis. Mol. Plant-Microbe Interact..

[35] Leiss K.A., Maltese F., Choi Y.H., Verpoorte R., Klinkhamer P.G. (2009). Identification of chlorogenic acid as a resistance factor for thrips in chrysanthemum. Plant Physiol..

[36] Stout M.J., Workman K.V., Bostock R.M., Duffey S.S. (1998). Stimulation and attenuation of induced resistance by elicitors and inhibitors of chemical induction in tomato (*Lycopersicon esculentum*) foliage. Entomol. Exp. Appl..

[37] Machado R.A., Ferrieri A.P., Robert C.A., Glauser G., Kallenbach M., Baldwin I.T., Erb M. (2013). Leaf-herbivore attack reduces carbon reserves and regrowth from the roots via jasmonate and auxin signaling. New Phytol..

[38] Schäfer M., Brütting C., Baldwin I.T., Kallenbach M. (2016). High throughput quantification of more than 100 primary- and secondary metabolites, and phytohormones by a single solid-phase extraction based sample preparation with analysis by UHPLC-HESI-MS/MS. Plant Methods.

[39] Leiss K.A., Cristofori G., van Steenis R., Verpoorte R., Klinkhamer P.G.L. (2013). An eco-metabolomic study of host plant resistance to Western flower thrips in cultivated, biofortified and wild carrots. Phytochemistry.

[40] Dara S.K. (2019). The new integrated pest management paradigm for the modern age. J. Integr. Pest Manag..

[41] Cantrell C.L., Dayan F.E., Duke S.O. (2012). Natural products as source for new pesticides. J. Nat. Prod..

[42] Lorsbach B.A., Sparks T.C., Cicchillo R.M., Garizi N.V., Hahn D.R., Meyer K.G. (2019). Natural Products: A Strategic Lead Generation Approach in Crop Protection Discovery. Pest Manag. Sci..

[43] Buitenhuis R., Brownbridge M., Brommit A., Saito T., Murphy G. (2016). How to start with a clean crop: Biopesticide dips reduce populations of *Bemisia tabaci* (Hemiptera: Aleyrodidae) on greenhouse poinsettia propagative cuttings. Insects.

[44] Krauter P.C., Heinz K.M., Arthurs S. (2017). Protecting unrooted cuttings from *Bemisia tabaci* (Hemiptera Aleyrodidae) during propagation. J. Insect Sci..

[45] Zhao Y. (2018). Essential roles of local auxin biosynthesis in plant development and in adaptation to environmental changes. Annu. Rev. Plant Biol..

[46] Felton G.W. (2005). Indigestion is a plant’s best defense. Proc. Natl. Acad. Sci. USA.

[47] Conrath U., Beckers G.J.M., Flors V., García-Agustín P., Jakab G., Mauch F., Newman M.A., Pieterse C.M.J., Poinssot B., Pozo M.J. (2006). Priming: Getting ready for battle. Mol. Plant Microbe Interact..

[48] Huot B., Yao J., Montgomery B.L., He S.Y. (2014). Growth-defense tradeoffs in plants: A balancing act to optimize fitness. Mol. Plant.

[49] Zhang W., Fan J., Tan Q., Zhao M., Zhou T., Cao F. (2017). The effects of exogenous hormones on rooting process and the activities of key enzymes of *Malus hupehensis* stem cuttings. PLoS ONE.

[50] Yan S.P., Yang R.H., Wang F., Sun L.N., Song X.S. (2017). Effect of auxins and associated metabolic changes on cuttings of hybrid aspen. Forests.

[51] International Agency for Research on Cancer (2010). Carbon black, titanium dioxide, and talc. IARC Working Group on the Evaluation of Carcinogenic Risks to Humans.

[52] Huberty A.F., Denno R.F. (2004). Plant water stress and its consequences for herbivorous insects: A new synthesis. Ecology.

[53] Thaler J.S., Bostock R.M. (2004). Interactions between abscisic-acid-mediated responses and plant resistance to pathogens and insects. Ecology.

[54] Erb M., Köllner T., Degenhardt J., Zwahlen C., Hibbard B.E., Turlings T.C. (2011). The role of abscisic acid and water stress in root herbivore-induced leaf resistance. New Phytol..

[55] Ellis C.M., Nagpal P., Young J.C., Hagen G., Guilfoyle T.J., Reed J.W. (2005). AUXIN RESPONSE FACTOR1 and AUXIN RESPONSE FACTOR2 regulate senescence and floral organ abscission in *Arabidopsis thaliana*. Development.

[56] Zhang Y., Wang H.L., Li Z., Guo H. (2020). Genetic Network between Leaf Senescence and Plant Immunity: Crucial Regulatory Nodes and New Insights. Plants.

