# Supplementary material for: Water Dipping of Auxin Coated Chrysanthemum Cuttings Confers Protection against Insect Herbivores
Source: Insects. 2020 Nov 12;11(11):790. doi: 10.3390/insects11110790 (PMC7697673; doi:10.3390/insects11110790)
Supplement: Supplementary file 1 [file insects-11-00790-s001.pdf]

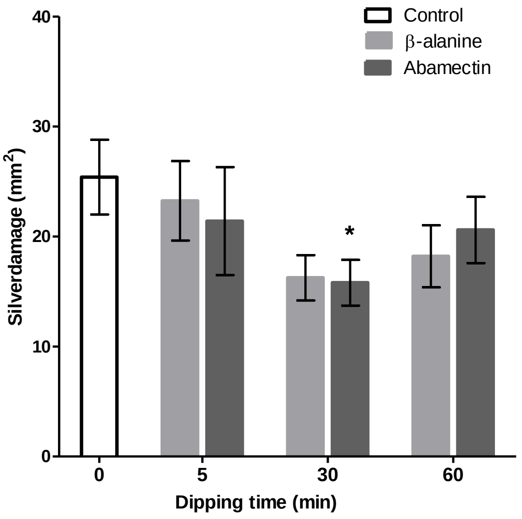

Figure S1. Efficacy of bio-insecticidal dips on chrysanthemum resistance against western flower thrips (WFT). The basal cut ends were dipped in 100 mg/ml of β-alanine or 0.3 ml/L abamectin (Avis EC, Syngenta, Syngenta Crop protection Inc., Greensboro, NC, USA) for various time points. Untreated, non-dipped, cuttings served as control. Two weeks post treatment, cuttings were infested with 20 adult thrips. One week after thrips infestation, silver damage symptoms were visually scored and expressed as damaged leaf area in mm^2. Data represent cumulative silver damage and are presented as mean ± SEM of 5 replicates per treatment. Asterisks indicate significant difference at α = 0.05 as determined by an unpaired Student's t-test.

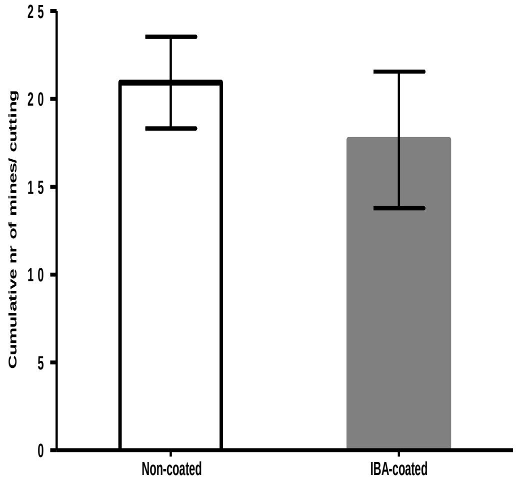

(**a**)

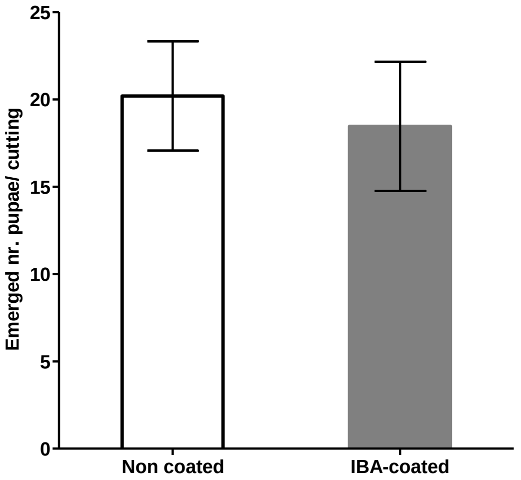

(**b**)

Figure S2. Effect of exogenously applied indole-3-butyric acid (IBA) on celery leaf miner (*Liriomyza trifolii*) resistance. Basal ends of chrysanthemum cuttings (cv. Baltica) were pre-coated with powder formulated rooting hormone (Chryzotek beige 0.4% IBA). Non-coated cuttings served as control. After 14 days of rooting, cuttings were infested with four one-day old leafminers (2 males and 2 females) for 24 h. (**a**) cumulative number of individual mines and (**b**) the number of emerged pupae. Data represent means ± SEM of 15 replicates. Differences between IBA-coated cuttings and control plants were evaluated by the Mann-Whitney U test (two-tailed) at $p < 0.05$.

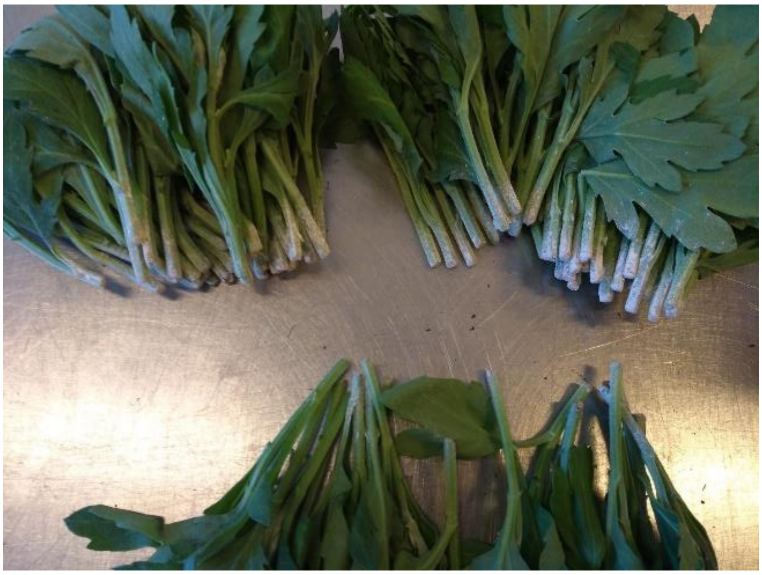

Figure S3. Substantial variation in the amount of hormone applied rooting powder within a batch of 50 commercially provided Baltica cuttings. Cuttings at the left upper part of the image contain considerably more rooting powder than the lower part whereas, cuttings on the left upper part are loaded with a mediocre amount. Approximately 1 cm of the basal cut end is pre-coated with the rooting powder Chryzoteck beige 0.4% (Rhizopon, Hazerswoude-Rijndijk, The Netherlands) consisting of 0.4% indole-3 butyric acid (IBA) in talc. Baltica cuttings require IBA applied rooting hormones for quick root initiation.

Table S1. Transitions or specific pair of m/z values associated to the precursors and fragment ions of the analytes measured by LC/MS.

Analyte	Q1 (m/z) → Q3 (m/z) [a]	CE (V)
**ABA**	(−) 263.13 → 153.0	9
**JA**	(−) 209.07 → 59.0	12
**JA-Ile**	(−) 322.2→ 130.1	19
**SA**	(−) 137.02 → 93.0	15
**IAA**	(+) 176.00 → 130.00	14
**D6-ABA**	(−) 269.0 → 159.0	9
**D6-JA**	(−) 215.15 → 59.0	10
**D6-JA-Ile**	(−) 328.2 → 130.0	19
**D4-SA**	(−) 141.05 → 97.0	15
**D6-IAA**	(−)141.05 → 97.00	15
**D5-IAA** [b]	(+) 181.00 → 135.00	14
	(+) 181.10 → 134.00	14
	(+) 181.10 → 133.00	14
**OPDA**	(−) 291.5 → 165.0	15

Note: Abbreviations: CE, collision energy. [a] Resolution Q1: 0.7, Q3: 22; [b] Analyzed as the sum of all three transitions.

Table S2. Backward multiple regression models of independent predictors of variation in silver damage.

Model	Independent Variables	B	SE	β	*t*	*p*
1	OPDA	0.049	0.055	0.212	0.891	0.396
	JA-ile	−22.390	10.812	−0.561	−2.071	0.068
	JA	0.183	0.094	0.583	1.951	0.083
	IAA	−0.054	0.111	−0.149	−0.484	0.640

	ABA	0.163	0.137	0.301	1.190	0.265
	SA	0.065	0.090	0.184	0.720	0.490
2	OPDA	0.045	0.052	0.194	0.860	0.410
	JA-ile	−23.247	10.249	−0.583	−2.268	0.047
	JA	0.158	0.075	0.504	2.099	0.062
	ABA	0.143	0.126	0.264	1.140	0.281
	SA	0.066	0.087	0.186	0.760	0.465
3	OPDA	0.048	0.051	0.210	0.955	0.360
	JA-ile	−19.522	8.827	−0.489	−2.212	0.049
	JA	0.146	0.072	0.465	2.021	0.068
	ABA	0.146	0.123	0.269	1.184	0.261
4	JA-ile	−19.950	8.783	−0.500	−2.271	0.042
	JA	0.159	0.070	0.507	2.253	0.044
	ABA	0.113	0.118	0.208	0.956	0.358
5	JA-ile	−21.308	8.639	−0.534	−2.466	0.028
	JA	0.176	0.068	0.562	2.594	0.022

Note: Independent variables: concentration of 12-oxo-phytodienoic acid (OPDA), jasmonic acid-isoleucine (JA-Ile), jasmonic acid (JA), indole-3-acetic acid (IAA), abscisic acid (ABA) and salicylic acid (SA). Abbreviations: B: unstandardized regression coefficient, β: standardized regression coefficient, SE: standard error Linear regression through the origin at $p < 0.05$.

Table S3. Model summary of the backward elimination multiple regression analysis.

**Model** [a]	**Adj R^2**	**df 1**	**df 2**	**F**	**Sig.**
1	0.257	6	9	1.865	0.192 [b]
2	0.314	5	10	2.374	0.115 [c]
3	0.340	4	11	2.935	0.071 [d]
4	0.345	3	12	3.636	0.045 [e]
5	0.350	2	13	5.030	0.024 [f]

Note: Abbreviations: Adj, adjusted; df, degrees of freedom. * $p < 0.01$ (two-tailed). [a] Dependent variable: silver damage. [b] Predictors: (Constant), SA, IAA, OPDA, ABA, JA-ile, JA; [c] Predictors: (Constant), SA, OPDA, ABA, JA-ile, JA; [d] Predictors: (Constant), OPDA, ABA, JA-ile, JA [e] Predictors: (Constant), ABA, JA-ile, JA; [f] Predictors: (Constant), JA-ile, JA.
